# Human papillomavirus oncogenic E6 protein regulates human β-defensin 3 (hBD3) expression via the tumor suppressor protein p53

**DOI:** 10.18632/oncotarget.8443

**Published:** 2016-03-28

**Authors:** Twishasri DasGupta, Emeka I. Nweze, Hong Yue, Liming Wang, Jessica Jin, Santosh K. Ghosh, Hameem I. Kawsar, Chad Zender, Elliot J. Androphy, Aaron Weinberg, Thomas S. McCormick, Ge Jin

**Affiliations:** ^1^ Department of Biological Sciences, Case Western Reserve University School of Dental Medicine, Cleveland, OH, USA; ^2^ Department of Otolaryngology-Head & Neck Surgery, University Hospitals Case Medical Center, Case Western Reserve University School of Medicine, Cleveland, OH, USA; ^3^ Department of Dermatology, University Hospitals Case Medical Center, Case Western Reserve University School of Medicine, Cleveland, OH, USA; ^4^ Center for Molecular Cancer Diagnosis Inc., Twinsburg, OH, USA; ^5^ Human Developmental and Regenerative Biology, Harvard University, Cambridge, MA, USA; ^6^ Department of Dermatology, Indiana University School of Medicine, Indianapolis, IN, USA; ^7^ Present Address: St. Luke's Hospital, Chesterfield, MO, USA; ^8^ Present Address: University of Nigeria, Nsukka, Nigera

**Keywords:** human papillomavirus, human β-defensins, p53, head and neck cancer, microenvironment

## Abstract

Human β-defensin-3 (hBD3) is an epithelial cell-derived innate immune regulatory molecule overexpressed in oral dysplastic lesions and fosters a tumor-promoting microenvironment. Expression of hBD3 is induced by the epidermal growth factor receptor signaling pathway. Here we describe a novel pathway through which the high-risk human papillomavirus type-16 (HPV-16) oncoprotein E6 induces hBD3 expression in mucosal keratinocytes. Ablation of E6 by siRNA induces the tumor suppressor p53 and diminishes hBD3 in HPV-16 positive CaSki cervical cancer cells and UM-SCC-104 head and neck cancer cells. Malignant cells in HPV-16-associated oropharyngeal cancer overexpress hBD3. HPV-16 E6 induces hBD3 mRNA expression, peptide production and gene promoter activity in mucosal keratinocytes. Reduction of cellular levels of p53 stimulates hBD3 expression, while activation of p53 by doxorubicin inhibits its expression in primary oral keratinocytes and CaSki cells, suggesting that p53 represses hBD3 expression. A p53 binding site in the hBD3 gene promoter has been identified by using electrophoretic mobility shift assays and chromatin immunoprecipitation (ChIP). In addition, the p63 protein isoform ΔNp63α, but not TAp63, stimulated transactivation of the hBD3 gene and was co-expressed with hBD3 in head and neck cancer specimens. Therefore, high-risk HPV E6 oncoproteins may stimulate hBD3 expression in tumor cells to facilitate tumorigenesis of HPV-associated head and neck cancer.

## INTRODUCTION

Human β-defensins (hBDs) are small cationic peptides originally identified from the plasma of patients with renal disease and from psoriatic skin lesions as innate antimicrobial molecules [1]. We have reported that proliferating oral mucosal basal layer cells as well as tumor cells in carcinoma in situ lesions and at the invasive front of squamous cell carcinoma produce hBD3, which is encoded by the *DEFB103A* gene, with little-to-no expression of hBD-1 and -2 [2, 3]. However, differentiated epithelial cells of oral epithelia only express hBD-1 and -2 [2, 3]. Tumor cell-derived hBD3 is associated with recruitment and activation of tumor-associated macrophages (TAMs) in the tumor microenvironment, thus contributing to tumor progression [2, 3]. We have reported that hBD3 expression is induced by activation of epidermal growth factor receptor (EGFR) [2]. The EGFR signaling pathway is also critical for hBD3 gene expression in cells following microbial insults [4]. Cetuximab (Erbitux), a humanized monoclonal antibody against EGFR, blocks LPS-induced hBD3 expression, indicating the importance of EGFR in modulation of hBD3 gene expression [4].

Human papillomaviruses (HPV) are non-lytic, non-enveloped viruses containing a small (~ 8 kbp), double-strand circular DNA genome encoding 6 early (E) and 2 late (L) proteins [5]. HPV infection by high-risk HPV types, particularly HPV-16 and -18, predisposes women to cervical cancer and acts as independent predictors for increased risk for the development of anal, penile, and vulvar cancers [5, 6]. Recent studies have also revealed a causal link between chronic HPV infection and a subset of head and neck squamous cell carcinomas (HNSCC), particularly tumors that arise largely from the lingual and palatine tonsils in the oropharynx [7]. HPV-16 is the most commonly detected HPV DNA in HNSCC, accounting for up to 90-95% of all HPV-associated HNSCC [7–9]. High-risk HPV E6 protein has been demonstrated to complex with the cellular ubiquitin ligase E6AP and p53, resulting in ubiquitination and degradation of the tumor suppressor p53 [10]. E7 binds to all members of the retinoblastoma (*RB*) gene family, leading to release of the repressive function and up-regulation of proteins involved in cell cycle progression [10]. Collectively, the oncogenic capacity of E6 and E7 proteins allows for maintenance of proliferation and inappropriate centrosomal and chromosomal duplication; frequently leading to malignant transformation [10].

Although oncogenic E6 and/or E7 proteins lead to reduced production of monocyte chemoattractant protein-1 (MCP-1) and other chemokines [11], TAMs still infiltrate HPV-16-associated tumors, resulting in suppression of an antitumor T cell response, thereby facilitating tumor growth [12]. Expression of human β-defensins and other antimicrobial peptides has been detected in HPV-associated vulvovaginal lesions and anal intraepithelial neoplasia [13, 14]. However, the production of hBD3 in HPV-associated HNSCC and the role of HPV oncoproteins in modulating hBD3 expression are still unknown. In the current study, we report that (1) clinically confirmed HPV-positive head and neck cancers overexpress hBD3, (2) HPV-16 E6 induces hBD3 expression in human oral epithelial cells and oral cancer cell lines when compared to E6 from non-oncogenic HPV types, and (3) this induction appears to be inversely regulated by p53 and induced by ΔNp63α. Our study therefore supports hBD3 as a novel HPV regulated factor that facilitates HPV-associated tumor development and progression.

## RESULTS

### Expression of hBD3 in HPV-positive cancer cells

**Figure 1:**
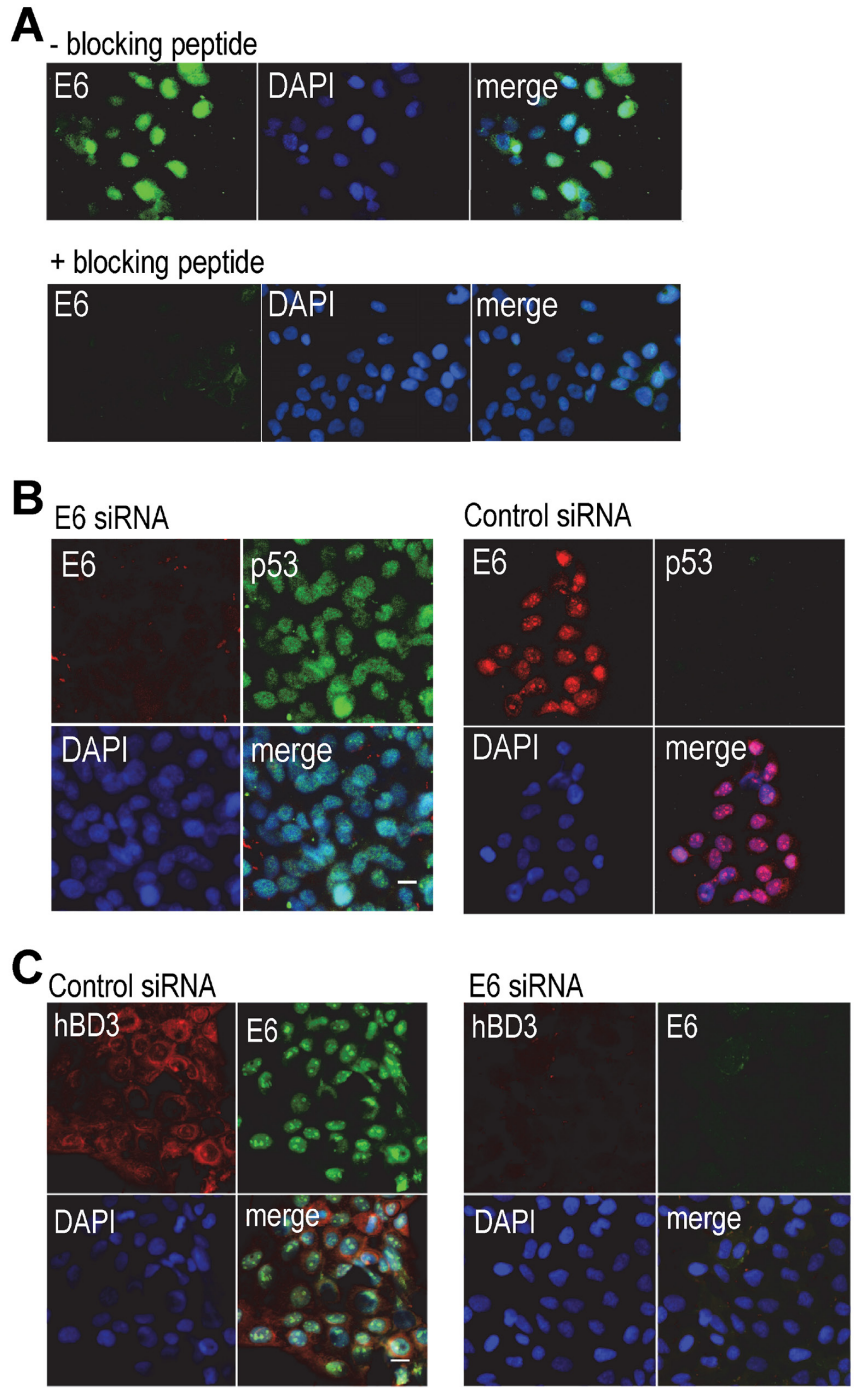
Association of hBD3 expression with HPV-16 E6 in CaSki cells **A.** CaSki cells were stained with the goat polyclonal antibody to HPV-16 E6 (upper panels, - blocking peptide) or the antibody pre-incubated with the blocking peptide (+ blocking peptide). E6, green; nuclei, blue (DAPI). 20x. **B.** CaSki cells were transfected with the E6 (left panel) or a control siRNA (right panels), followed by immunofluorescent staining of HPV-16 E6 (red) and p53 (green). Nuclei, blue (DAPI). 20x. **C.** CaSki cells were transfected with a control or the E6 siRNA and then stained for hBD3 (red) and HPV-16 E6 (green). Nuclei, blue (DAPI). 20x.

We tested specificity of commercially available HPV-16 E6 antibodies by performing immunofluorescence microscopy on HPV-positive CaSki cervical cancer cells, which contain multiple copies of integrated HPV-16 genome. The goat polyclonal antibody against HPV-16 E6 (N-17) (Santa Cruz Biotech Inc.) showed nuclear E6 staining in CaSki cells (Figure 1A), but not in that of HPV-negative TR146 oral cancer cells (Supplementary Figure S1). Addition of the blocking peptide to the N-17 antibody eliminated E6 staining (Figure 1A). The goat IgG isotype control antibody did not show this fluorescence signal in CaSki cells (Supplementary Figure S1). In addition, a mouse monoclonal antibody to HPV-16/18 E6 (C1P5, Santa Cruz Biotech Inc.) only showed cytoplasmic staining (Supplementary Figure S1). It has been shown that reduction of HPV gene expression by HPV-16 E6 siRNA is associated with increase in the level of nuclear p53 in CaSki cells [15]. To further validate the goat anti-HPV-16 E6 antibody, CaSki cells were transfected with an HPV-16 E6 siRNA or a control siRNA, followed by immunofluorescence microscopy for HPV-16 E6 and p53. The polyclonal HPV-16 E6 antibody did not detect the E6 protein in CaSki cells transfected with the E6 siRNA, while the monoclonal antibody to p53 identified nuclear p53 protein in the cells (Figure 1B). In addition, control siRNA had no effect on nuclear staining of E6 with undetectable p53 in CaSki cells (Figure 1B). To determine hBD3 expression in HPV-positive cancer cells, we performed immunofluorescent double-staining on CaSki cells using a polyclonal antibody to hBD3 and the antibody against E6. CaSki cells expressed hBD3 in the cytoplasm with co-expression of nuclear E6 in cells transfected with the control siRNA (Figure 1C, left panel). However, transfection of CaSki cells with the E6 siRNA blocked hBD3 as well as nuclear E6 expression (Figure 1C, right panel). These results suggest association of hBD3 expression with E6 in HPV-positive cancer cells.

### Expression of hBD3 in HPV-positive head and neck (HNC) cancer

**Figure 2:**
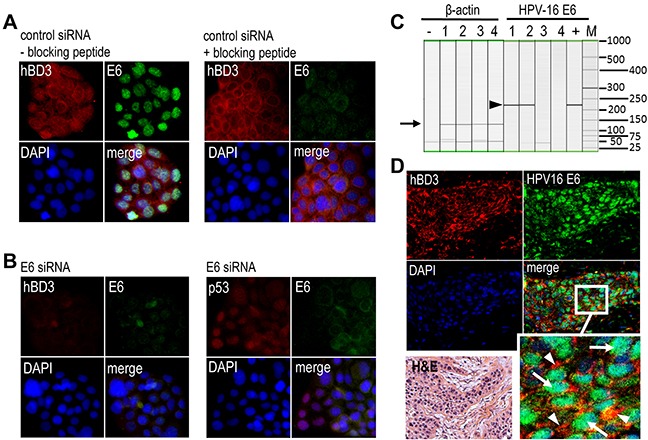
Expression of hBD3 in HPV-16 positive oropharyngeal cancer specimens **A.** UM-SCC-104 cells grown in cell culture glass slides were transfected with control siRNA and then stained with the goat polyclonal antibody to HPV-16 E6 in the absence (right panels, - blocking peptide) or presence of the blocking peptide (right panels, +blocking peptide). hBD3, red; E6, green; nuclei, blue (DAPI). 20x. **B.** UM-SCC-104 cells were transfected with the E6 siRNA, followed by immunofluorescent staining of hBD3 (red) and HPV-16 E6 (green, left panel) as well as p53 (red) and HPV-16 E6 (green, right panel). Nuclei, blue. 20x. **C.** Multiplex high-risk HPV genotyping on head and neck cancer genome DNA. 1, 2, oropharyngeal cancer biopsies; 3, 4, oral squamous cell carcinoma biopsies; +, a positive control DNA derived from a cervical cancer patient specimen with confirmed HPV-16; M, DNA molecular weight markers. Only HPV-16 (arrow head) genotyping and β-actin genomic DNA (arrow) gel images are shown. **D.** Immunofluorescence microscopy of hBD3 (red, arrowheads in enlarged inset) and HPV-16 E6 (green, arrows in enlarged inset). H&E; H&E stain from the same specimen. 20x.

High-risk HPV infection is a causal factor for a subset of head and neck cancer, particularly those arising from the lingual and palatine tonsils in the oropharynx [7]. We have demonstrated that hBD3 overexpression in head and neck neoplastic cells is associated with accumulation and activation of tumor-associated macrophages and may facilitate cancer progression [3]. To determine if HPV-associated head and neck cancer produce hBD3 and the role of HPV oncoproteins in modulating hBD3 expression, HPV-16-positive UM-SCC-104 head and neck cancer cells [16] were transfected with HPV-16 E6 or a control siRNA, followed by immunofluorescence microscopy for hBD3 and E6. Control siRNA transfected cells showed cytoplasmic hBD3 and nuclear E6 expression (Figure 2A, left panel). The blocking peptide of the HPV-16 E6 antibody eliminated E6 staining, but not hBD3, in UM-SCC-104 cells (Figure 2A, right panel). Transfection of cells with the E6 siRNA blocked expression of both E6 and hBD3 (Figure 2B, left panel), but induced that of p53, in UM-104-SCC cells (Figure 2B, right panel), suggesting the association of hBD3 expression with HPV-16 E6 in HPV-positive HNSCC cells. To evaluate the expression of hBD3 in HPV-associated HNSCC specimens, we first genotyped high-risk HPVs using a multiplex PCR approach by amplifying E6 and E7 for each of 13 high-risk HPV types, 16, 18, 31, 33, 35, 39, 45, 51, 52, 56, 58, 59 and 68, on genomic DNA extracted from 4 HNC specimens. Two oropharyngeal cancer samples were found to be HPV-16 E6 positive with a specific 223 bp band, while two oral squamous cell carcinoma (OSCC) samples were HPV-16 E6 negative (Figure 2C). We did not detect any other high-risk HPVs in these oropharyngeal cancer specimens. To ensure the quality of the genomic DNA, a 133 bp region of the β-actin gene in all genomic DNA samples was amplified with predicted size in all samples (Figure 2C). We then determined whether HPV-16 E6 and hBD3 could be detected in biopsy specimens by using immunofluorescence microscopy. Immunofluorescence images show high levels of hBD3 in the cytoplasm of cancer cells co-expressing HPV-16 E6 protein in their nuclei (Figure 2D). This phenotype of co-localization of cytoplasmic hBD3 and nuclear HPV-16 E6 suggests that cancer cells of HPV-associated HNSCC overexpress hBD3.

### Induction of hBD3 gene expression by HPV-16 E6

**Figure 3:**
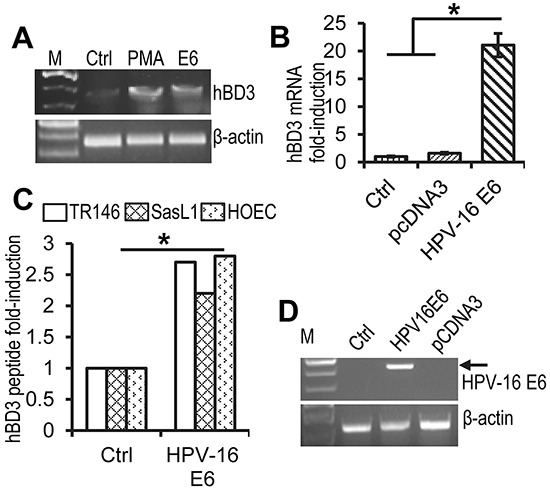
Induction of hBD3 expression by HPV-16 E6 **A.** RT-PCR for hBD3 and human β-actin mRNA on total RNA extracted from OKFT6/TERT-2 cells that were transfected with the HPV16 E6 expression construct. PMA, phorbol 12-myristate-13-acetate (PMA) treated cells, positive controls; Ctrl, mock transfection control. **B.** Fold induction of hBD3 mRNA in HOECs transfected with the HPV-16 E6 expression construct. Cells transfected with the pcDNA3 vector (pcDNA3) and mock transfection (Ctrl) were used as negative controls. *, p≤ 0.05 (n=3). **C.** HOECs and SasL1 and TR146 oral cancer cells were transfected with the HPV-16 E6 expression construct for 48 hr, followed by hBD3 ELISA of cell lysates. Production of hBD3 peptide was presented as fold-increase of hBD3 produced by cells transfected with the HPV-16 E6 expression construct compared with those transfected with pcDNA3. *, p≤ 0.05 (n=3). **D.** RT-PCR of total RNA derived from HEK293 cells transfected with HPV-16 E6, or a pcDNA3 vector. Ctrl, mock transfected cells.

To determine whether HPV-16 E6 directly regulates hBD3 expression, immortalized OKF6/TERT-2 human oral keratinocytes were transiently transfected with the HPV-16 E6 expression construct, followed by RT-PCR of hBD3 mRNA. The expression of hBD3 mRNA was induced by HPV-16 E6 in OKF6/TERT-2 cells comparably to those treated with PMA, a known inducer of hBD3 [2] (Figure 3A). Transfection of primary human oral epithelial cells (HOECs) [2] with the HPV-16 E6 expression construct also induced hBD3 mRNA by over 30-fold as measured by real-time quantitative RT-PCR (qRT-PCR) (Figure 3B). In addition, HPV-16 E6 induced production of hBD3 peptide in HOECs and two HPV-negative oral cancer cell lines SasL1 and TR146 as determined by hBD3 ELISA using cell lysates (Figure 3C). HPV E6 expression undergoes alternative splicing to generate various transcripts that have been identified in cervical cancer cell lines and HPV-16 immortalized keratinocytes [17]. Transcription from the early viral promoter p97 generates E6, E6f*1, and E6*2 mRNA species that can be detected by RT-PCR in several cervical cancer cell lines [17]. To determine whether these spliced forms were expressed in transient transfection of the HPV-16 E6 expression construct, we performed RT-PCR on total RNA isolated from HOECs transfected with the HPV-16 E6 expression vector using primers that covered the full length of the E6 transcript. The RT-PCR only resulted in a single band that represented un-spliced mRNA species of HPV-16 E6 (Figure 3D), indicating that transient transfection of the HPV-16 E6 gene does not generate alternative splicing transcripts and that un-spliced E6 is responsible for inducing hBD3.

### Transcriptional regulation of hBD3 gene expression by HPV-16 E6 and the effect of low- and high-risk E6 on hBD3 expression

**Figure 4:**
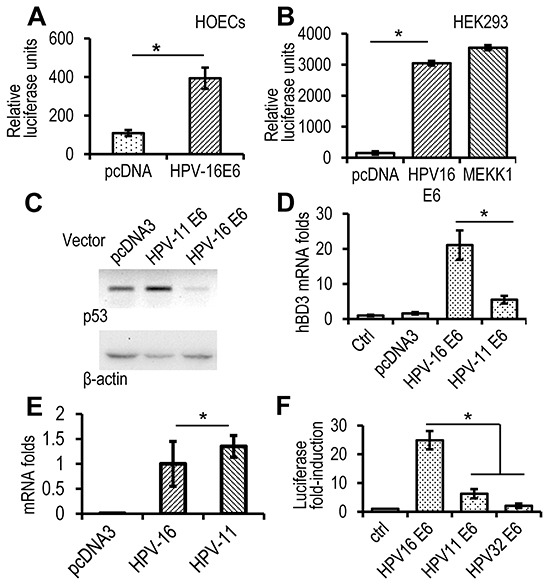
High-risk, but not low-risk, HPV E6 induces expression of hBD3 **A.** Luciferase analysis in HOECs transfected with HPV-16 E6 expression construct or a blank pcDNA3 vector together with the hBD3 luciferase reporter. Relative luciferase units represent hBD3 promoter activity after normalized with the Renilla luciferase activity using the dual-luciferase system. *, p≤ 0.05 (n=3). **B.** Luciferase analysis in HEK293 cells transfected with HPV-16 E6, or an MEKK expression construct together with the hBD3 luciferase reporter. *, p≤ 0.05 (n=3). **C.** Western blotting on p53 protein in HEK293 cells transfected with E6 expression construct of HPV-16 or -11. **D.** Real-time qRT-PCR of HOECs transfected with E6 expression construct of HPV-16 or -11. *, p≤ 0.05 (n=3). **E.** Real-time qRT-PCR of E6 mRNA of HPV-16 and -11 in HOECs transfected with respective expression constructs. Transfection with the blank pcDNA3 vector was used as a negative control. *, p≥ 0.56. **F.** hBD3 gene promoter reporter analysis in HOECs transfected with E6 expression construct of HPV-16, -11, or -32. *, p≤ 0.05 (n=3). Luciferase fold-induction indicates relative luciferase units of cells transfected with expression constructs compared to that transfected with the blank pcDNA3 vector (Ctrl).

To identify the HPV-16 responsive elements in the hBD3 gene promoter, HOECs were transfected with a reporter construct that contains a ~1.5 kbp promoter region of the hBD3 gene driving the expression of firefly luciferase, together with the HPV-16 E6 expression construct for measurements of the reporter luciferase activity. HPV-16 E6 promoted luciferase activity significantly higher than the blank pcDNA3 vector (Figure 4A), indicating that E6 induces expression of hBD3 at the transcriptional level. HEK293 cells have been used for investigating interactions of ectopic proteins with p53, since the cell line contains wild-type p53 and serves as a transfection host for exogenous gene expression [15, 18]. Transfection of the HPV-16 E6 expression vector produced E6 in HEK293 cells by western blotting (Supplementary Figure S2). As in HOECs, HPV-16 E6 significantly induced activation of the hBD3 gene promoter, comparable to that caused by the MEKK1 expression construct [2], in HEK293 cells (Figure 4B).

High-risk HPV E6 proteins induce degradation of the tumor suppressor p53 protein through the E3 ubiquitin ligase activity of E6AP and, together with the E7 oncoprotein, promotes carcinogenesis. However, low-risk E6 proteins, such as those found in HPV-11, do not degrade p53 [19]. Therefore, we questioned whether E6 proteins of high- and low-risk HPVs would differentially modulate hBD3 expression. HPV-16 E6, but not that of HPV-11 E6, diminished p53 in HEK293 cells (Figure 4C). HPV-16 E6 induced significantly higher levels of hBD3 mRNA than HPV-11 E6 in HOECs (Figure 4D); while expression levels of these E6 genes were the same (Figure 4E). Similarly, HPV-16 E6, but not HPV-11 or HPV-32 E6, another low-risk species, induced significant activation of the hBD3 gene promoter in HEK293 cells (Figure 4F). These results suggested that reduction of the p53 protein was associated with the induction of hBD3 expression.

### Modulation of hBD3 expression by the tumor suppressor protein p53

**Figure 5:**
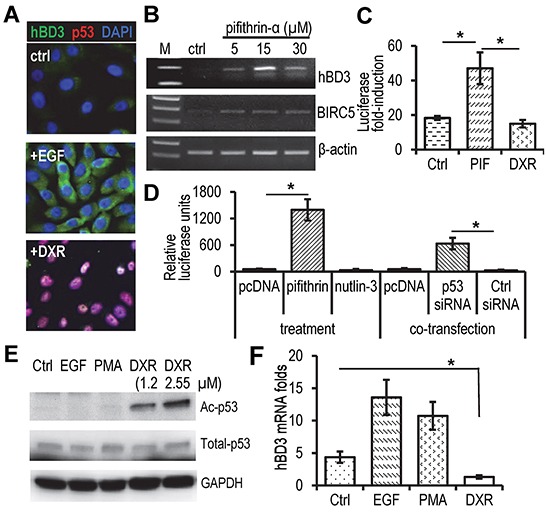
Effect of p53 on hBD3 expression **A.** HOEC cells were treated with EGF (10 ng/ml) or doxorubicin (DXR, 1.25 μM) for 16 hr, followed by immunofluorescent staining with antibodies to hBD3 (green) and p53 (red). Nuclei, blue (DAPI). 20x. **B.** Effect of the p53 inhibitor pifithrin-α on hBD3 and BIRC5 (survivin) expression in HOECs using RT-PCR. **C.** Effect of pifithrin-α (PIF) and DXR on the promoter activity of the hBD3 gene in HOECs. **D.** Effect of p53 inhibitor (pifithrin-α), activator (nutlin-3) and siRNA targeting p53 on transactivation of the hBD3 gene promoter in HEK293 cells. The assay was conducted in triplicates and repeated three times. *, p≤ 0.05 (n=3). **E.** Western blotting of acetylated p53 (Ac-p53), total p53, and GAPDH in CaSki cells treated with EGF (20 ng/ml), PMA (5 nM), and DXR at different concentrations for 16 hr. **F.** qRT-PCR of hBD3 mRNA in CaSki cells treated with DXR (2.5 μM), EGF (20 ng/ml), and PMA (5 nM) for 16 hr. Ctrl, no treatment. *, p≤ 0.05. The experiment was repeated 3 times.

We proposed that p53 would repress hBD3 gene expression. Indeed, HOECs produced hBD3 without visible p53 protein in response to EGF treatment as shown by immunofluorescence microscopy analysis (Figure 5A). Treatment of HOECs with Doxorubicin (DXR), an anthracycline antibiotic that up-regulates and activates p53 [20], induced expression and nuclear translocation of p53, concomitantly inhibiting production of hBD3 (Figure 5A). In addition, inactivation of p53 by pifithrin-α, a small molecular agent that inhibits transcriptional activity of p53 [21], induced mRNA expression of hBD3 and BIRC5 (Survivin), a protein known to be repressed by p53 [22] (Figure 5B). While pifithrin-α stimulated the activity of the hBD3 gene promoter, DXR blocked it in HOECs (Figure 5C). Pifithrin-α and p53 siRNA also enhanced hBD3 promoter activity in HEK293 cells (Figure 5D). However, treating cells with nutlin-3, which enhances accumulation of p53 by antagonizing the p53 modulatory protein MDM2 [23], did not induce promoter activity (Figure 5D). Acetylation plays a positive role in the accumulation and transcriptional activation of p53 protein in stress response [24]. HPV-positive CaSki cervical cancer cells contain wild-type p53, which can be acetylated in response to DXR treatment [25]. To determine the expression profile of hBD3 and its association with p53 in CaSki cells, we treated the cells with DXR, EGF, and PMA. DXR induced acetylation of p53, while EGF and PMA had no effect on its protein levels and acetylation (Figure 5E). Accordingly, hBD3 expression was significantly reduced in CaSki cells treated with DXR compared with those treated with EGF, PMA, or remained un-treated (Ctrl) (Figure 5F). Our results suggest that p53 functions as a transcriptional repressor of hBD3 gene expression and that HPV-16 E6 can modulate hBD3 expression in a p53-dependent mechanism.

### Binding of p53 to the hBD3 gene promoter and induction of hBD3 gene transactivation by ΔNp63

**Figure 6:**
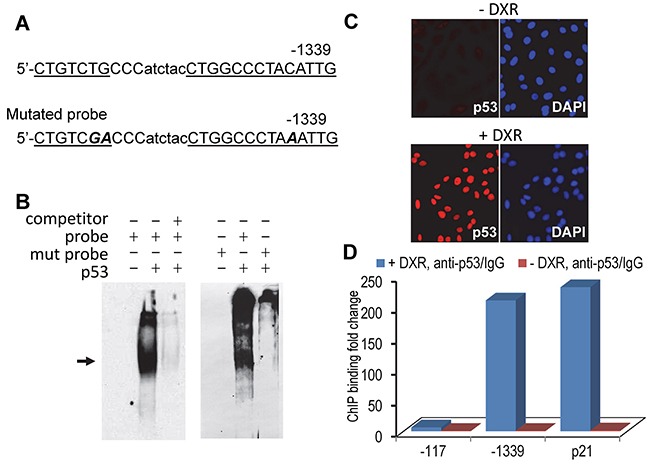
Identification of p53 binding site in the hBD3 gene promoter **A.** Putative p53-binding site in the hBD3 promoter identified by computational analysis. The p53 responsive element was underlined. Mutant oligonucleotides in the p53 responsive sequence are marked as bold/italicized letters. **B.** EMSA of recombinant p53 protein with hBD3 promoter derived, biotin-labeled oligonucleotides (probe) as shown in A. Shifted band is indicated by an arrow. Un-labeled oligonucleotides were used as competitors to ensure the specificity of the binding. Mutated probe (mut probe) failed to induce shifted p53/probe complex. **C.** OKF6/TERT-2 cells were treated with doxorubicin followed by immunofluorescent staining for p53 (red). Nuclei, blue (DAPI). 20x. **D.** ChIP analysis of p53 interaction with the hBD-3 promoter in vivo. OKF6/TERT-2 cells treated with or without DXR (+/− DXR) were used in ChIP assays using qPCR. The abundance of p53 binding sites was represented as the ratio of DNA derived from p53 antibody precipitation (anti-p53) vs. that from control IgG incubation (IgG). -1339, the putative p53 binding site in the hBD3 gene promoter; -179, the site of a genomic DNA fragment without p53 binding; p21, ChIP on the p53 binding site in the p21 gene. The experiment was repeated two times.

p53 functions as a transcription factor by directly binding to promoter sequences or via indirect protein-protein interaction. Our data showed that p53 repressed hBD3 expression at the transcriptional level; we therefore postulated that p53 directly bound to p53 response element(s) of the hBD3 gene promoter, resulting in inhibition of the hBD3 promoter activity. To identify putative p53 binding sites in the hBD3 promoter, the 1.5 kbp hBD3 gene promoter region was analyzed by PROMO, a virtual laboratory for the prediction of putative transcription factor binding sites in DNA sequences based on the TRANSFAC database [26]. Computational analysis identified several putative p53 binding sites. To determine p53 binding to the putative responsive elements within the DNA probe as identified by the program, electrophoretic mobility shift assays (EMSA) were performed using purified recombinant p53 protein with biotin-labeled oligonucleotide probes. One such putative binding sequence, starting at -1339 upstream of the transcription start site (Figure 6A), bound to purified recombinant p53 protein (Figure 6B). Binding was abolished by addition of excess amount of un-labeled oligonucleotides (Figure 6B). To further confirm that the probe contained p53 responsive elements, we generated a mutant probe with three nucleotide mutations (Figure 6A) for EMSA. The mutant probe failed to bind to the recombinant p53 protein to induce the shifted band (Figure 6B), indicating that the p53 responsive element contains sequences 5′- CTGTCTGCCC and 5′-CCCTACATTGG in the hBD3 gene promoter. To determine the presence of p53 in the hBD3 promoter in vivo, chromatin immunoprecipitation (ChIP) assays were conducted using OKF6/TERT2 cells, which were treated with 1.25 μm of DXR for 5 hr to induce p53 (Figure 6C), followed by crosslinking of protein-chromatin complexes by formalin and precipitation of the complexes using the monoclonal antibody to human p53. ChIP data showed that DXR induced enrichment of the p53-chromatin complexes by about 225-fold at the p53 binding site located at -1339 bp, compared to cells without DXR treatment, indicating that p53 physically binds to the hBD3 promoter in vivo (Figure 6D). In the ChIP assay, the binding of p53 to the p53 responsive element of the p21 gene was also identified and served as a positive control (Figure 6D).

**Figure 7:**
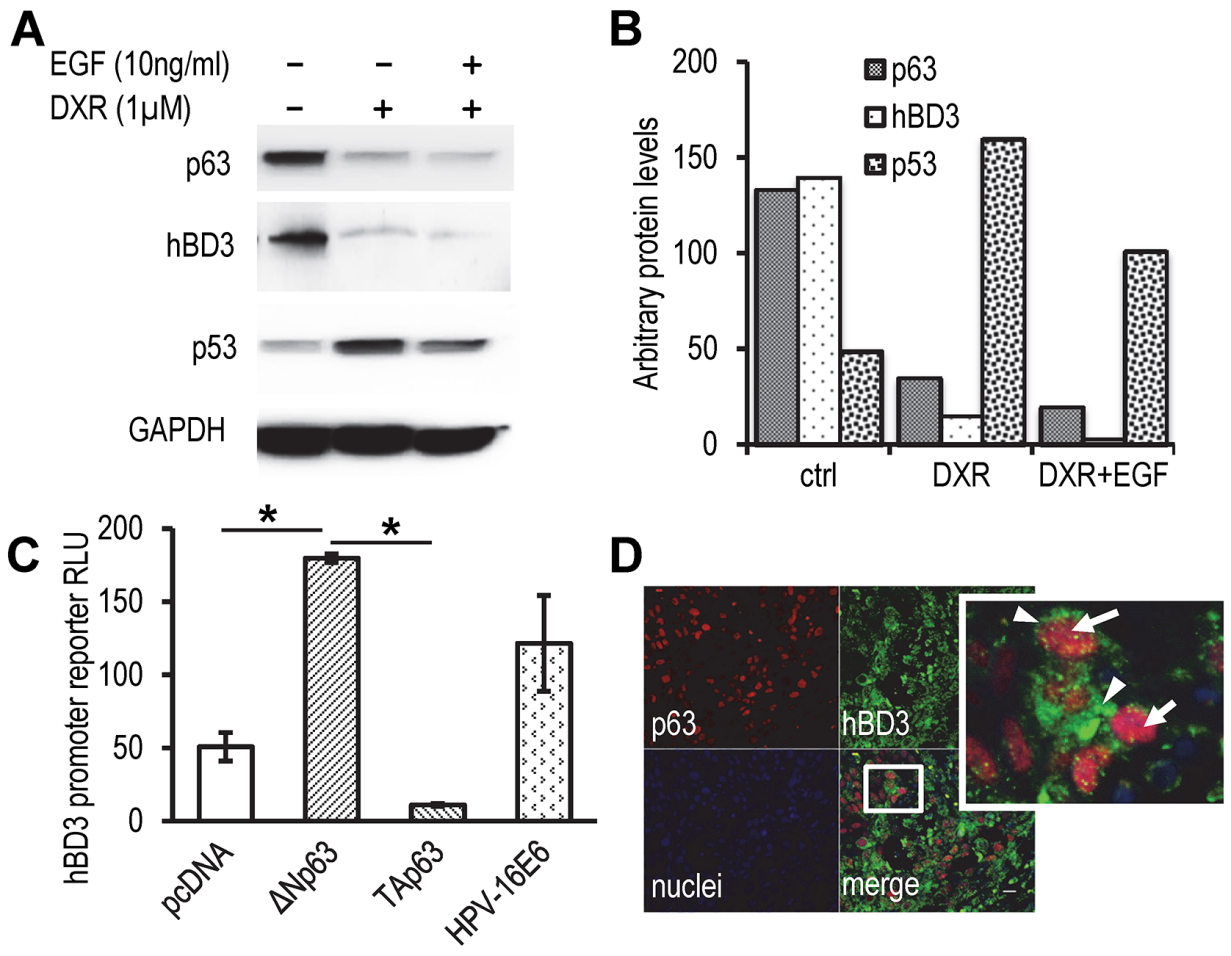
Role of p63 in modulation of hBD3 expression **A.** HOECs were grown in basal medium with supplements, following addition of EGF (10 ng/ml) and DXR (1 μM) for 20 hr. Western blotting for p53, p63, and hBD3 was performed with GAPDH as a housekeeping control. **B.** Quantification of western blotting densities in A. **C.** Luciferase activities in HEK293 cells co-transfected with the hBD3 gene promoter reporter and expression constructs as indicated. RLU, relative luciferase units. pcDNA blank vector transfection was used as a negative control. Luciferase activities were determined using the Dual-luciferase system. *, p<0.05. **D.** Expression of hBD3 and p63 in HNSCC specimens using immunofluorescence microscopy. A representative image is shown. Arrows, p63 (red); arrow heads, hBD3 (green); nuclei, blue (DAPI). 20x.

As shown in Figure 3C, the E6 protein induced hBD3 expression in HOECs and the SasL1 HNSCC cell line containing a wild-type p53 [27, 28]. However, E6 protein also stimulated hBD3 expression in TR146 HNSCC cells that have a disruptive p53 gene [29, 30], suggesting that E6 can modulate hBD3 gene expression in a p53-indipendent fashion. The p53 paralogue, p63 protein, has been shown to partially overlap promoter-binding activity with p53 [31]. p63 is encoded by the *TP63* gene to produce the full-length TAp63, which contains the N-terminal transactivation (TA) domains, and the ΔNp63 isoforms, which lack TA domains and are deficient in transactivation [31]. p63 participates in modulating expression of several p53 repressive genes, including *CD44* and *BIRC5* [32, 33]. To evaluate the effect of p63 on regulation of hBD3 expression, HOECs were grown in keratinocyte culture medium supplemented with EGF treated with DXR or with DXR plus excess amount of EGF, followed by western blotting of total p63, p53, and hBD3 proteins. We found that p63 and hBD3 proteins in HOECs grown in control culture medium were significantly higher compared to those in cells treated with DXR with or without excess amount of EGF (Figure 7A). However, DXR significantly induced p53 protein in HOECs (Figure 7A). The expression pattern of p63 and hBD3 proteins vs. that of p53 in HOECs were quantified (Figure 7B). Since the antibody to p63 in the western blotting recognizes all isoforms of p63, we decided to evaluate whether TAp63 or ΔNp63α was involved in modulation of hBD3 gene expression using promoter reporter assays of the hBD3 gene. We found that ΔNp63α, but not TAp63, induced activity of the hBD3 gene promoter (Figure 7C). To assess the association of hBD3 and p63 in patient samples, HNSCC biopsy specimens were stained for hBD3 and p63 and showed concomitant expression of hBD3 and p63 in cancer cells (Figure 7D). Our results indicate that ΔNp63α, in contrast to p53, induces hBD3 expression and that HPV-16 E6 may modulate hBD3 gene expression in a p53-independent mechanism, probably via activation of p63.

## DISCUSSION

Recent epidemiological and molecular studies have determined that HPV-associated head and neck cancers are on the rise [7, 34–36]. TAMs have been shown to significantly enhance the development and progression of HPV-related cancers by releasing tumor-promoting cytokines, chemokines, and growth factors into the tumor microenvironment [12, 37, 38]. Migration of macrophages to the anatomical sites of tumors has been described to be mediated by MCP-1/CCL2 in a variety of cancers [39]. However, MCP-1 expressing tumor cells in head and neck cancer are rare and not correlated with macrophage migration in biopsy samples of these cancers [2]. We reported that oral carcinoma in situ lesions overexpress hBD3 with little-to-no expression of MCP-1 [2, 3] and that tumor cell-derived hBD3 is associated with accumulation of TAMs in the lesion site [3]. Furthermore, hBD3 induces migration of human and mouse monocytes via the chemokine receptor CCR2 [3], suggesting that hBD3 may contribute to the recruitment of TAMs [3]. In the present study, we first rigorously validated a polyclonal antibody using HPV-16 positive CaSki cervical cancer cells and then demonstrated that high-risk HPV E6 was co-localized with hBD3 in HPV-positive head and neck cancer tissues. In addition, transfection of cultured epithelial cells with the HPV-16 E6 expression vector induced hBD3 mRNA expression, suggesting that HPV-16 E6 may be the causal agent for hBD3 expression in HPV-positive head and neck cancer. Interestingly, E6 proteins of low-risk HPVs were not able to induce transactivation of the hBD3 promoter compared to that of HPV-16. Therefore, hBD3 induced by E6 of high-risk HPVs may facilitate accumulation and activation of TAMs in the tumor microenvironment, resulting in suppression of the acquired immune response to tumor cells, and thereby promote carcinogenesis in the head and neck region.

We have reported that EGF induces the expression of hBD3 in oral epithelial cells via MAP kinases p38 and JNK, PI3K/AKT, and PKC [2]. In addition, hBD3 is expressed in proliferating cell nuclear antigen (PCNA)-positive basal cells in normal oral mucosa and in tumor cells of oral carcinoma in situ lesions [2]. Herein, we focused on how expression of hBD3 was modulated by HPV-16 E6 protein. HPV-16 is responsible for the majority (~ 90% to 95%) of HPV-positive oropharyngeal squamous cell carcinomas [9], in which predominantly wild-type p53 is inactivated by E6. This inactivation occurs through two distinct mechanisms: (1) E6 protein associates with the cellular E6 association protein (E6AP, UBA3), an E3 ubiquitin ligase, to recruit p53 for degradation via the proteasome pathway, and (2) it also interacts with p300 and Ada3 to block p300-mediated p53 acetylation, an essential protein modification for p53 activation [40–42]. Our results showed that inactivation of p53 significantly induced hBD3 expression. However, activation of p53 suppressed hBD3 expression. These data indicate that p53 suppresses hBD3 gene expression.

Identification of a p53-regulated gene requires that three criteria be met; these include: (1) the presence of a p53 responsive element in the DNA close to or within the gene, (2) up- or down-regulation at the mRNA and protein levels as well as the gene promoter responses to p53, and (3) identification of p53 binding sequences in the gene promoter region by either ChIP assays or EMSA [43]. Recent studies indicate that a majority of p53-repressed genes are regulated by binding of p53 at the distal region of the genome to interfere with the activity of the enhancers of p53-repressed genes [44]. For example, in the absence of p53, transcription enhancers at the distal region of *Nanog*, one of the p53-repressed core transcription factors critical for pluripotency in mouse embryonic stem cells, contribute to activation of *Nanog* transcription. However, binding of p53 at the enhancers inhibits enhancer activity, resulting in repression of *Nanog* expression [44]. In this report, we have identified a potential p53 binding site at -1339 upstream of the transcription start site in the hBD3 promoter via EMSA and ChIP analysis. The fact that the p53 binding site is located in the hBD3 promoter suggests that p53 can repress hBD3 expression by directly binding to the p53 responsive elements of the hBD3 gene promoter.

The canonical p53 binding sites in the genome generally consist of two copies of the degenerated sequences 5′-PuPuPuC(A/T)-3′ arranged head-to-head and divided by a spacer of 0-21 base pairs [43]. The half-sites vary in size between 8 to 12 bp, but most have 10 bp [43]. Our results show that the p53 binding site in the hBD3 gene promoter contains two half-sites 5′-CTGTCGACCC and 5′-CCCTAAATTG separated in the head-to-tail arrangement, which has been reported in other p53-repressed genes, such as *ARF*, *CCNA*, and *CD44* [32, 45, 46]. Therefore, our date indicate that suppression of hBD3 expression by p53 uses the same mechanism.

p53 mutation is a frequent event in HPV-negative HNSCC [47, 48]. It has been shown that over 70% of HNSCC cases contain p53 mutations [49]. In this report, we have shown that transfection of HPV-16 E6 gene induced hBD3 protein expression in SasL1 and TR146 HNSCC cell lines (Figure 3C). SasL1 is a subline of the SAS tongue carcinoma cell line that contains a p53 with wild-phenotype [27, 28, 50], while the TR146 cell line, which was derived from a cervical lymph node metastasis that originated from a well-differentiated buccal HNSCC [51], has a homozygous mutation in exon 7 of the *TP53* gene [29, 30]. Our results showed that HPV-16 E6 induced expression of hBD3 mRNA in both wild-type and mutant p53 HNSCC cell lines, suggesting that HPV-16 E6 can induce hBD3 gene expression in a p53-dependent and p53-independent pathways. Various isoforms of p63 are highly expressed in the basal layers of epithelia and play important roles in stem cell homeostasis [52]. TAp63 can inhibit tumorigenesis and metastasis in vivo, while ΔNp63 isoforms, particularly ΔNp63α, is involved in tumorigenesis [53–55]. It has been shown that cervical squamous cell carcinomas over-produce p63 and that cervical carcinoma cell lines constitutively express ΔNp63α [56–58]. In addition, ΔNp63α is predominantly produced in undifferentiated normal human foreskin keratinocytes (NHKs) compared to differentiated NHKs. However, HPV E6 and E7 proteins cause retention of ΔNp63α expression in differentiated NHKs [59]. In addition, it has been shown that ΔNp63 and p53 antagonize with each other during tissue development and tumorigenesis [53]. Given the role of tumor cell-derived hBD3 in promoting tumorigenesis, our findings suggest that high-risk HPVs induce hBD3 expression through regulation of cellular p53 and ΔNp63α, a novel pathway that facilitates progression of HPV-associated cancers.

Taken together, our data have identified a novel mechanism by which high-risk HPV-16, through its well-known virulence factor E6, utilizes the suppression of p53, in the presence of ΔNp63α, to induce selective overexpression of hBD3; an epithelial cell derived antimicrobial and immunoregulatory peptide that, under normal conditions, acts to defend mucosal surfaces from microbial challenges. In the context of neoplasia, hBD3 may now be considered as a possible therapeutic target for the development of potential therapeutic approaches of head and neck cancer.

## MATERIALS AND METHODS

### Tissue samples, cell culture and reagents

Formalin-fixed, paraffin-embedded (FFPE) tissue sample protocols and waiver of informed consent as well as written informed consents and protocols using human oral keratinocytes were approved by Case Comprehensive Cancer Center and Case Western Reserve University Institutional Review Board, respectively. Primary human oral epithelial cells (referred as to HOECs thereafter) were isolated from healthy patients who underwent third-molar extraction at School of Dental Medicine as described [60]. HOECs and immortalized OKF6/TERT-2 human oral keratinocytes were maintained as previously described [2, 61]. HPV-16-positive UM-SCC-104 HNSCC cells ([16], purchased from EMD Millipore (Billerica, MA)), oral cancer cell lines SasL1 [28], TR146 [2], as well as adenovirus type 5 (Ad5) transformed human embryonic kidney epithelial (HEK293) cells were maintained in Dulbecco's Modified Eagle Media (DMEM) (Invitrogen, Carlsbad, CA) supplemented with 10% fetal bovine serum (FBS) and Anti-Anti antibiotics (Invitrogen). Antibodies used in the work were: goat polyclonal anti-HPV16 E6 (N-17) and anti-human β-defensin 3, mouse monoclonal anti-human p63 and -HPV-16/18 E6 (C1P5) (Santa Cruz Biotech., Santa Cruz, CA); mouse monoclonal anti-human p53 (Epitomics, Burlingame, CA); rabbit polyclonal anti-human p53 (Abcam, Cambridge, MA), rabbit polyclonal anti-human acetylated-p53 (K382) (Cell Signaling, Danvers, MA), rabbit polyclonal anti-hBD3 (Novus, Littleton, CO); Alexa Fluor-conjugated donkey monoclonal antibodies to IgGs of various species (Invitrogen). IgGs of mouse and goat were purchased from Invitrogen. The blocking peptide to the goat polyclonal antibody to HPV-16 E6 (N-17), HPV-16 E6 siRNA, p53 siRNA, and the control siRNA were purchased from Santa Cruz Biotech. Each of siRNA products generally consist of pools of three to five target-specific 19-25 nt siRNAs designed to knockdown gene expression based on the manufacturer's instructions (Santa Cruz Biotech). Phorbol 12-myristate-13-acetate (PMA), pifithrin-α, nutlin-3, and doxorubicin (DXR) were purchased from Sigma-Aldrich (St Louis, MO).

### HPV genotyping in formalin-fixed, paraffin-embedded (FFPE) biopsy specimens

Genomic DNA was extracted from 2-4 FFPE sections of HNSCC specimens using QIAamp DNA FFPE Tissue Kit (Qiagen Inc., Germantown, MD). High-risk HPV genotyping by PCR was run at 95°C, 5 min and then 45 cycles of 95°C for 30 sec., 56°C for 30 sec, followed by 72°C for 45 sec. In addition, PCR for a 133 bp β-actin gene fragment was used as positive control. PCR products with specific sizes were detected by capillary gel electrophoresis (Qiagen).

### Immunofluorescence microscopy and western blotting

Immunofluorescence microscopy of biopsy samples was performed as described before [2, 3]. Fluorescent micrographs were taken by the EVOS fluorescence microscope (Life Tech). Western blotting was performed using the Mini-PROTEAN with pre-cast gels following the manufacture's instruction (Bio-Rad, Hercules, CA). For hBD3 blotting, a 20% SDS-PAGE gel was used.

### RNA extraction, RT-PCR, and real-time quantitative PCR

Total RNA was extracted using GeneElute mammalian total RNA isolation kit (Sigma-Aldrich) and reverse-transcribed into the first strand cDNA using the SuperScript III reverse-transcriptase and hexamers (Invitrogen). Semi-quantification of PCR products [2] and real-time quantitative PCR on total RNA were performed as described [62]. The glyceraldehyde 3-phosphate dehydrogenase (GAPDH) cDNA was amplified as an endogenous reference. Amplification was performed at 40 cycles of 94°C for 15 s followed by 60°C for 1 m and quantified. Each qPCR was run in triplicates and the experiment was repeated at least 3 times. For real-time quantitative RT-PCR of HPV-16 and -11 of HPV-16 and -11 genes, the following primers were used (forward/reverse): HPV-16 E6, 5′-ATGCACCAAAAGAGAACTGCAA/5′ TCACACAACGGTTTGTTGTAT and HPV-11 E6, 5′-CT CCACGTCTGCAACATCCATAGAC/5′-TGCCTGTTGCT TAGAACTGCAAGGGA.

### Transfection and luciferase assays

Transfection of plasmids was performed using Lipofectamine 2000 (Invitrogen) as described [2]. For promoter luciferase reporter assays, 100 ng of the pRL-CMV plasmid (Promega) was co-transfected into cells, providing an internal control to normalize transfection efficiency among different plasmids and treatments. Promoter reporter activity was determined using the dual luciferase kit (Promega, Madison, WI) following the manufacture's protocol. Transfection and luciferase measurements were carried out in triplicates and the experiments were repeated at least three times. Transfection of siRNA was performed using Sigma-Aldrich's N-TER Nanoparticle siRNA Transfection System with 140 nM (CaSki cells) or 200 nM (UM-SCC-104 cells) siRNA following the manufacture's instruction.

### Chromatin-immunoprecipitation (ChIP) and electrophoretic mobility gel shift assays (EMSA)

For ChIP assays, OKF6/TERT-2 cells cultured in 100 mm plates were treated with doxorubicin (DXR) (1.25 μM) for 5 hr to activate p53. Cells without treatment were used as a control. ChIP assays were performed using the ChIP Assay Kit following the manufacture's instruction (Upstate Biotechnology, Lake Placid, NY). The mouse monoclonal antibody to human p53 (Epitomics) was use to precipitate the protein-DNA complexes. DNA was purified and subjected to qPCR with primers 5′-TGATTGAGCTCCACTCTTGGCTCA and 5′-AGGTGAGGGTGAAGTGGATGAGA. The abundance of p53 binding was calculated by 2^-ΔCt, where ΔCt was determined by subtraction of the Ct of DNA of p53 antibody precipitation from that of DNA from control IgG incubation. The ChIP assay was repeated two times. For EMSA, oligonucleotides were synthesized with biotin labeling on the 3′-end (Integrated DNA Tech., Coralville, IA) with the sequences: 5′-TGACTGTCTGCCCATCTACCTGGCCCTACAT-biotin and the mutant probe 5′-TGACTGTCGACCCATCTACCTGGCCCTAAATTG-biotin (mutant nucleotides are underlined). Recombinant p53 protein (100 ng) (Excellgen, Rockville, MD) was added to 100 nM annealed biotin labeled probes in a binding buffer and EMSA was performed as described [63]. The gels were then transferred to a Nylon membrane and the DNA/protein complexes were visualized by the LightShift Chemiluminescent EMSA Kit (Pierce Biotech, Rockford, IL).

### Statistics

Real-time quantitative RT-PCR and relative luciferase activity results of treatments were compared with those of respective controls. The data were subjected to two-tailed paired Student's t-test with two-sample equal variance for comparison of two groups. p≤0.05 was considered to be statistically significant. Data analyses were performed and graphs were generated using Minitab program (Minitab Inc.) and Excel 2010 (Microsoft, Seattle, WA).

## SUPPLEMENTARY FIGURES



## References

[1] Harder J, Bartels J, Christophers E, Schroder JM (2001). Isolation and characterization of human beta -defensin-3, a novel human inducible peptide antibiotic. J Biol Chem.

[2] Kawsar HI, Weinberg A, Hirsch SA, Venizelos A, Howell S, Jiang B, Jin G (2009). Overexpression of human beta-defensin-3 in oral dysplasia: potential role in macrophage trafficking. Oral Oncol.

[3] Jin G, Kawsar HI, Hirsch SA, Zeng C, Jia X, Feng Z, Ghosh SK, Zheng QY, Zhou A, McIntyre TM, Weinberg A (2010). An antimicrobial peptide regulates tumor-associated macrophage trafficking via the chemokine receptor CCR2, a model for tumorigenesis. PLoS One.

[4] Shuyi Y, Feng W, Jing T, Hongzhang H, Haiyan W, Pingping M, Liwu Z, Zwahlen RA, Hongyu Y (2011). Human beta-defensin-3 (hBD-3) upregulated by LPS via epidermal growth factor receptor (EGFR) signaling pathways to enhance lymphatic invasion of oral squamous cell carcinoma. Oral Surg Oral Med Oral Pathol Oral Radiol Endod.

[5] Fehrmann F, Laimins LA (2003). Human papillomaviruses: targeting differentiating epithelial cells for malignant transformation. Oncogene.

[6] Uronis HE, Bendell JC (2007). Anal cancer: an overview. Oncologist.

[7] Fakhry C, Gillison ML (2006). Clinical implications of human papillomavirus in head and neck cancers. J Clin Oncol.

[8] Syrjanen S (2007). Human papillomaviruses in head and neck carcinomas. N Engl J Med.

[9] D'souza G, Kreimer AR, Viscidi R, Pawlita M, Fakhry C, Koch WM, Westra WH, Gillison ML (2007). Case-control study of human papillomavirus and oropharyngeal cancer. N Engl J Med.

[10] McCance DJ (2005). Human papillomaviruses and cell signaling. Sci STKE.

[11] Kanodia S, Fahey LM, Kast WM (2007). Mechanisms used by human papillomaviruses to escape the host immune response. Curr Cancer Drug Targets.

[12] Lepique AP, Daghastanli KR, Cuccovia IM, Villa LL (2009). HPV16 tumor associated macrophages suppress antitumor T cell responses. Clin Cancer Res.

[13] Erhart W, Alkasi O, Brunke G, Wegener F, Maass N, Arnold N, Arlt A, Meinhold-Heerlein I (2011). Induction of human beta-defensins and psoriasin in vulvovaginal human papillomavirus-associated lesions. J Infect Dis.

[14] Kreuter A, Skrygan M, Gambichler T, Brockmeyer N, Stücker M, Herzler C, Potthoff A, Altmeyer P, Pfister H, Wieland U (2009). Human papillomavirus-associated induction of human β-defensins in anal intraepithelial neoplasia. British Journal of Dermatology.

[15] Koh DI, Han D, Ryu H, Choi WI, Jeon BN, Kim MK, Kim Y, Kim JY, Parry L, Clarke AR, Reynolds AB, Hur MW (2014). KAISO, a critical regulator of p53-mediated transcription of CDKN1A and apoptotic genes. Proc Natl Acad Sci U S A.

[16] Tang AL, Hauff SJ, Owen JH, Graham MP, Czerwinski MJ, Park JJ, Walline H, Papagerakis S, Stoerker J, McHugh JB, Chepeha DB, Bradford CR, Carey TE, Prince ME (2012). UM-SCC-104: a new human papillomavirus-16-positive cancer stem cell-containing head and neck squamous cell carcinoma cell line. Head Neck.

[17] Rosenberger S, De-Castro Arce J, Langbein L, Steenbergen RD, Rosl F (2010). Alternative splicing of human papillomavirus type-16 E6/E6* early mRNA is coupled to EGF signaling via Erk1/2 activation. Proc Natl Acad Sci U S A.

[18] Thomas P, Smart TG (2005). HEK293 cell line: a vehicle for the expression of recombinant proteins. J Pharmacol Toxicol Methods.

[19] Brimer N, Lyons C, Vande Pol SB (2007). Association of E6AP (UBE3A) with human papillomavirus type 11 E6 protein. Virology.

[20] Minotti G, Menna P, Salvatorelli E, Cairo G, Gianni L (2004). Anthracyclines: molecular advances and pharmacologic developments in antitumor activity and cardiotoxicity. Pharmacol Rev.

[21] Gudkov AV, Komarova EA (2005). Prospective therapeutic applications of p53 inhibitors. Biochem Biophys Res Commun.

[22] Hoffman WH, Biade S, Zilfou JT, Chen J, Murphy M (2002). Transcriptional repression of the anti-apoptotic survivin gene by wild type p53. J Biol Chem.

[23] Vassilev LT, Vu BT, Graves B, Carvajal D, Podlaski F, Filipovic Z, Kong N, Kammlott U, Lukacs C, Klein C, Fotouhi N, Liu EA (2004). In vivo activation of the p53 pathway by small-molecule antagonists of MDM2. Science.

[24] Reed SM, Quelle DE (2014). p53 Acetylation: Regulation and Consequences. Cancers (Basel).

[25] Lee SJ, Hwang SO, Noh EJ, Kim DU, Nam M, Kim JH, Nam JH, Hoe KL (2014). Transactivation of bad by vorinostat-induced acetylated p53 enhances doxorubicin-induced cytotoxicity in cervical cancer cells. Exp Mol Med.

[26] Messeguer X, Escudero R, Farre D, Nunez O, Martinez J, Alba MM (2002). PROMO: detection of known transcription regulatory elements using species-tailored searches. Bioinformatics.

[27] Okumura K, Konishi A, Tanaka M, Kanazawa M, Kogawa K, Niitsu Y (1996). Establishment of high- and low-invasion clones derived for a human tongue squamous-cell carcinoma cell line SAS. J Cancer Res Clin Oncol.

[28] Muramatsu H, Kogawa K, Tanaka M, Okumura K, Nishihori Y, Koike K, Kuga T, Niitsu Y (1995). Superoxide dismutase in SAS human tongue carcinoma cell line is a factor defining invasiveness and cell motility. Cancer Res.

[29] Eicher SA, Clayman GL, Liu TJ, Shillitoe EJ, Storthz KA, Roth JA, Lotan R (1996). Evaluation of topical gene therapy for head and neck squamous cell carcinoma in an organotypic model. Clin Cancer Res.

[30] Skinner HD, Sandulache VC, Ow TJ, Meyn RE, Yordy JS, Beadle BM, Fitzgerald AL, Giri U, Ang KK, Myers JN (2012). TP53 disruptive mutations lead to head and neck cancer treatment failure through inhibition of radiation-induced senescence. Clin Cancer Res.

[31] Su X, Chakravarti D, Flores ER (2013). p63 steps into the limelight: crucial roles in the suppression of tumorigenesis and metastasis. Nat Rev Cancer.

[32] Godar S, Ince TA, Bell GW, Feldser D, Donaher JL, Bergh J, Liu A, Miu K, Watnick RS, Reinhardt F, McAllister SS, Jacks T, Weinberg RA (2008). Growth-inhibitory and tumor- suppressive functions of p53 depend on its repression of CD44 expression. Cell.

[33] Park HR, Min SK, Cho HD, Kim KH, Shin HS, Park YE (2004). Expression profiles of p63, p53, survivin, and hTERT in skin tumors. J Cutan Pathol.

[34] Grulich AE, Jin F, Conway EL, Stein AN, Hocking J (2010). Cancers attributable to human papillomavirus infection. Sex Health.

[35] Scudellari M (2013). HPV: Sex, cancer and a virus. Nature.

[36] Lingen MW, Xiao W, Schmitt A, Jiang B, Pickard R, Kreinbrink P, Perez-Ordonez B, Jordan RC, Gillison ML (2013). Low etiologic fraction for high-risk human papillomavirus in oral cavity squamous cell carcinomas. Oral Oncol.

[37] Ruffell B, Affara NI, Coussens LM (2012). Differential macrophage programming in the tumor microenvironment. Trends Immunol.

[38] Leemans CR, Braakhuis BJ, Brakenhoff RH (2011). The molecular biology of head and neck cancer. Nat Rev Cancer.

[39] Pollard JW (2004). Tumour-educated macrophages promote tumour progression and metastasis. Nat Rev Cancer.

[40] Talis AL, Huibregtse JM, Howley PM (1998). The role of E6AP in the regulation of p53 protein levels in human papillomavirus (HPV)-positive and HPV-negative cells. J Biol Chem.

[41] Patel D, Huang SM, Baglia LA, McCance DJ (1999). The E6 protein of human papillomavirus type 16 binds to and inhibits co-activation by CBP and p300. EMBO J.

[42] Kumar A, Zhao Y, Meng G, Zeng M, Srinivasan S, Delmolino LM, Gao Q, Dimri G, Weber GF, Wazer DE, Band H, Band V (2002). Human papillomavirus oncoprotein E6 inactivates the transcriptional coactivator human ADA3. Mol Cell Biol.

[43] Riley T, Sontag E, Chen P, Levine A (2008). Transcriptional control of human p53-regulated genes. Nat Rev Mol Cell Biol.

[44] Li M, He Y, Dubois W, Wu X, Shi J, Huang J (2012). Distinct regulatory mechanisms and functions for p53-activated and p53-repressed DNA damage response genes in embryonic stem cells. Mol Cell.

[45] Robertson KD, Jones PA (1998). The human ARF cell cycle regulatory gene promoter is a CpG island which can be silenced by DNA methylation and down-regulated by wild-type p53. Mol Cell Biol.

[46] Desdouets C, Ory C, Matesic G, Soussi T, Brechot C, Sobczak-Thepot J (1996). ATF/CREB site mediated transcriptional activation and p53 dependent repression of the cyclin A promoter. FEBS Lett.

[47] Rothenberg SM, Ellisen LW (2012). The molecular pathogenesis of head and neck squamous cell carcinoma. J Clin Invest.

[48] Molinolo AA, Amornphimoltham P, Squarize CH, Castilho RM, Patel V, Gutkind JS (2009). Dysregulated molecular networks in head and neck carcinogenesis. Oral Oncol.

[49] Iglesias-Bartolome R, Martin D, Gutkind JS (2013). Exploiting the head and neck cancer oncogenome: widespread PI3K-mTOR pathway alterations and novel molecular targets. Cancer Discov.

[50] Masunaga S, Uto Y, Nagasawa H, Hori H, Nagata K, Suzuki M, Kinashi Y, Ono K (2006). Evaluation of hypoxic cell radio-sensitizers in terms of radio-sensitizing and repair-inhibiting potential. Dependency on p53 status of tumor cells and the effects on intratumor quiescent cells. Anticancer Res.

[51] Rupniak HT, Rowlatt C, Lane EB, Steele JG, Trejdosiewicz LK, Laskiewicz B, Povey S, Hill BT (1985). Characteristics of four new human cell lines derived from squamous cell carcinomas of the head and neck. J Natl Cancer Inst.

[52] Alexandrova EM, Petrenko O, Nemajerova A, Romano RA, Sinha S, Moll UM (2013). DeltaNp63 regulates select routes of reprogramming via multiple mechanisms. Cell Death Differ.

[53] Yang A, Kaghad M, Wang Y, Gillett E, Fleming MD, Dotsch V, Andrews NC, Caput D, McKeon F (1998). p63, a p53 homolog at 3q27-29, encodes multiple products with transactivating, death-inducing, and dominant-negative activities. Mol Cell.

[54] Keyes WM, Pecoraro M, Aranda V, Vernersson-Lindahl E, Li W, Vogel H, Guo X, Garcia EL, Michurina TV, Enikolopov G, Muthuswamy SK, Mills AA (2011). DeltaNp63alpha is an oncogene that targets chromatin remodeler Lsh to drive skin stem cell proliferation and tumorigenesis. Cell Stem Cell.

[55] Bid HK, Roberts RD, Cam M, Audino A, Kurmasheva RT, Lin J, Houghton PJ, Cam H (2014). DeltaNp63 promotes pediatric neuroblastoma and osteosarcoma by regulating tumor angiogenesis. Cancer Res.

[56] Wang TY, Chen BF, Yang YC, Chen H, Wang Y, Cviko A, Quade BJ, Sun D, Yang A, McKeon FD, Crum CP (2001). Histologic and immunophenotypic classification of cervical carcinomas by expression of the p53 homologue p63: a study of 250 cases. Hum Pathol.

[57] Shirendeb U, Hishikawa Y, Moriyama S, Win N, Thu MM, Mar KS, Khatanbaatar G, Masuzaki H, Koji T (2009). Human papillomavirus infection and its possible correlation with p63 expression in cervical cancer in Japan, Mongolia, and Myanmar. Acta Histochem Cytochem.

[58] Ben Khalifa Y, Teissier S, Tan MK, Phan QT, Daynac M, Wong WQ, Thierry F (2011). The human papillomavirus E6 oncogene represses a cell adhesion pathway and disrupts focal adhesion through degradation of TAp63beta upon transformation. PLoS Pathog.

[59] Melar-New M, Laimins LA (2010). Human papillomaviruses modulate expression of microRNA 203 upon epithelial differentiation to control levels of p63 proteins. J Virol.

[60] Krisanaprakornkit S, Kimball JR, Weinberg A, Darveau RP, Bainbridge BW, Dale BA (2000). Inducible expression of human beta-defensin 2 by Fusobacterium nucleatum in oral epithelial cells: multiple signaling pathways and role of commensal bacteria in innate immunity and the epithelial barrier. Infect Immun.

[61] Dickson MA, Hahn WC, Ino Y, Ronfard V, Wu JY, Weinberg RA, Louis DN, Li FP, Rheinwald JG (2000). Human keratinocytes that express hTERT and also bypass a p16(INK4a)-enforced mechanism that limits life span become immortal yet retain normal growth and differentiation characteristics. Mol Cell Biol.

[62] Quinones-Mateu ME, Lederman MM, Feng Z, Chakraborty B, Weber J, Rangel HR, Marotta ML, Mirza M, Jiang B, Kiser P, Medvik K, Sieg SF, Weinberg A (2003). Human epithelial beta-defensins 2 and 3 inhibit HIV-1 replication. Aids.

[63] Jin G, Klika A, Callahan M, Faga B, Danzig J, Jiang Z, Li X, Stark GR, Harrington J, Sherf B (2004). Identification of a human NF-kappaB-activating protein, TAB3. Proc Natl Acad Sci U S A.

