# Supplementary material for: Human papillomavirus oncogenic E6 protein regulates human β-defensin 3 (hBD3) expression via the tumor suppressor protein p53
Source: Oncotarget. 2016 Mar 28;7(19):27430–44. doi: 10.18632/oncotarget.8443 (PMC5053661; doi:10.18632/oncotarget.8443)
Supplement: Supplementary file 1 [file oncotarget-07-27430-s001.pdf]

SUPPLEMENTARY FIGURES

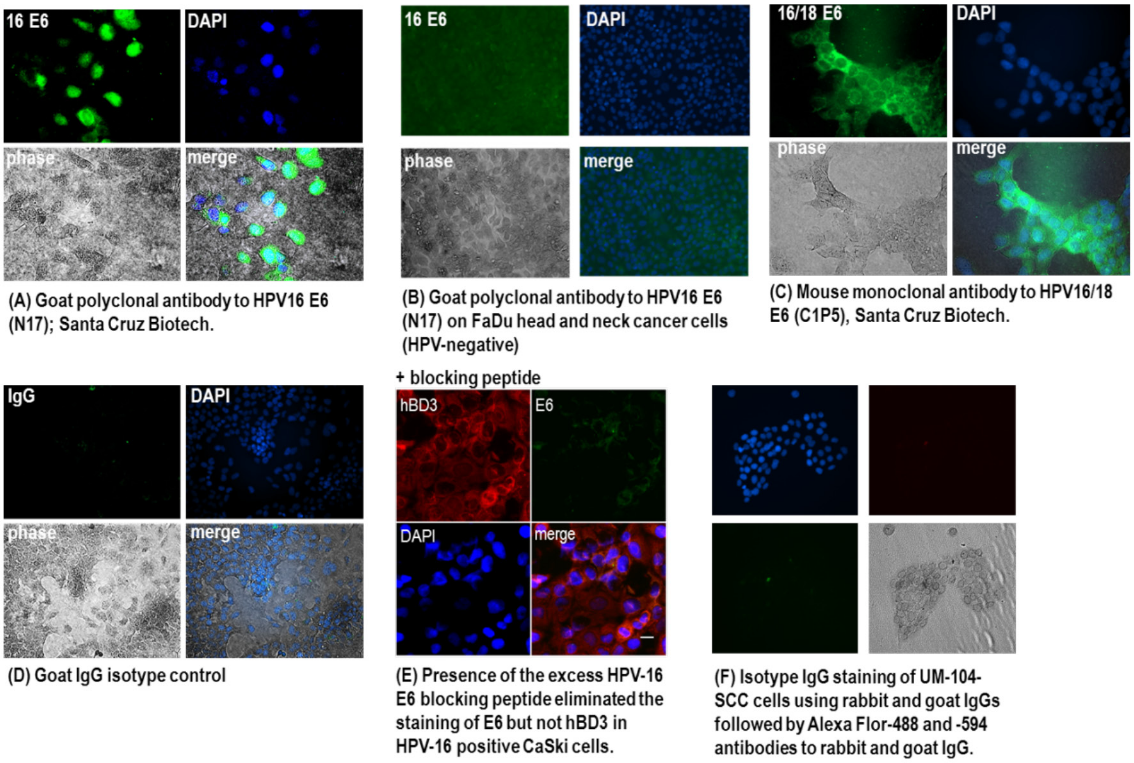

Supplemetary Figure S1: Validation of antibodies to HPV16 oncoproteins in CaSki cells. A. The goat polyclonal antibody to HPV-16 E6 (N-17, Santa Cruz Biotech Inc.) was used to stain HPV-16-postivei CaSki cells. HPV-16 E6, green; nuclei, blue (DAPI); 20x. **B.** Immunofluorescent staining of HPV-negative TR146 oral cancer cells with the HPV-16 E6 polyclonal antibody (N-17, Santa Cruz Biotech Inc.). **C.** Immunofluorescent staining of CaSki cells using the mouse monoclonal antibody that can detect E6 of both HPV-16 and -18 (Santa Cruz Biotech Inc.). 20x. **D.** CaSki cells isotype control. **E.** Effect of excess HPV-16 E6 antibody blocking peptide on staining of hBD3 (red) and E6 (green). Blue, nuclei (DAPI). 20x. **F.** Isotype IgG control staining of UM-SCC-104 cells. Cells were incubated with goat and rabbit IgG proteins overnight at 4°C, followed by staining using Alexa Fluor-488 anti-goat IgG and Alex Fluor-596 anti-rabbit IgG antibodies.

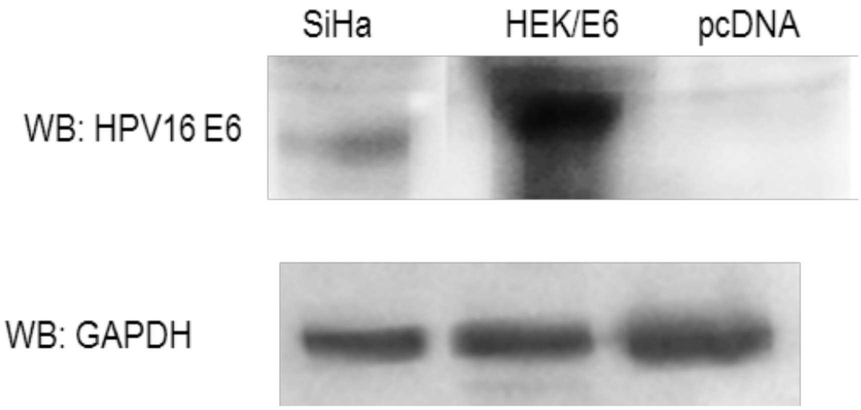

Supplemetary Figure S2: Western blotting of HPV-16 E6 on cell lysates of HEK293 cells transfected with the HPV-16 E6 expression construct. HPV-16-positive SiHa cervical cancer cells were used as a positive control.
